# Self-other voice discrimination task: A potential neuropsychological tool for clinical assessment of self-related deficits

**DOI:** 10.1016/j.heliyon.2024.e38711

**Published:** 2024-10-02

**Authors:** Philippe Voruz, Pavo Orepic, Selim Yahia Coll, Julien Haemmerli, Olaf Blanke, Julie Anne Péron, Karl Schaller, Giannina Rita Iannotti

**Affiliations:** a Department of Neurosurgery, University Hospitals of Geneva, 1205, Geneva, Switzerland; b Clinical and Experimental Neuropsychology Laboratory, Faculty of Psychology, University of Geneva, 1205, Geneva, Switzerland; c Department of Basic Neurosciences, Faculty of Medicine, University of Geneva, 1202, Geneva, Switzerland; d Laboratory of Cognitive Neurorehabilitation, Faculty of Medicine, University of Geneva, 1205, Geneva, Switzerland; e Laboratory of Cognitive Neuroscience, Neuro-X Institute and Brain Mind Institute, Faculty of Life Sciences, Swiss Federal Institute of Technology (EPFL), 1211, Geneva, Switzerland; f NeuroCentre, University Hospitals of Geneva, 1205, Geneva, Switzerland

## Abstract

**Background:**

Deficits in self are commonly described through different neuro-pathologies, based on clinical evaluations and experimental paradigms. However, currently available approaches lack appropriate clinical validation, making objective evaluation and discrimination of self-related deficits challenging.

**Methods:**

We applied a statistical standardized method to assess the clinical discriminatory capacity of a Self-Other Voice Discrimination (SOVD) task. This task, validated experimentally as a marker for self-related deficits, was administered to 17 patients eligible for neurosurgery due to focal hemispheric brain tumors or epileptic lesions.

**Results:**

The clinical discriminatory capacity of the SOVD task was evident in three patients who exhibited impairments for self-voice perception that could not be predicted by other neuropsychological deficits. Impairments in other-voice perception were linked to inhibitory neuropsychological deficits, suggesting a potential association with executive deficits in voice recognition.

**Conclusions:**

This exploratory study highlights the clinical discriminatory potential of the SOVD task and suggests that it could complement the standard neuropsychological assessment, paving the way for enhanced diagnoses and tailored treatments for self-related deficits.

## Highlights

• Evaluating self-voice allows us to observe self-deficits.
• Validation of an experimental task with psychometric measures.
• Voice assessment could be integrated into clinical neuropsychology.

## 1 Introduction

The assessment of self-related deficits, conceptually related in the neurological and neuropsychological literature, to the terms anosognosia, unconsciousness, unconcern and anosodiaphoria [1,2] is of great importance in clinical care whether in the context of acquired neurological pathologies (e.g., traumatic brain injuries [3], stroke [4]), neurodegenerative diseases (e.g., Alzheimer's disease [5], Parkinson's disease [6]), neuroimmunological disorders (e.g., multiple sclerosis [7]) and post-infectious syndromes (e.g., post-COVID-19 condition [8]), as well as before and following interventions (e.g., neurosurgical [9]) revealing the transdiagnostic implications of this symptom. In addition, self-related deficits can significantly impact other neuropsychological functions, particularly their association with executive functioning and their implications for neurorehabilitation processes, as well as activities of daily living. Due to the current lack of dedicated investigation, it is crucial to develop and integrate specific measures for comprehensive patient management [1,2]. However, the current understanding and diagnosis of self-related deficits are constrained by the lack of clinical tests that are psychometrically validated as standard neuropsychological tools. Indeed, the standard neuropsychological assessments of self-related deficits rely on the clinician's subjective evaluation, questionnaires, or a comparison between objective performance and the patient's subjective evaluation [4,5]. In this context, an objective evaluation derived from experimental paradigms investigating the self, based on psychometrical analysis (i.e. quantitative values) adapted to clinical practice (i.e. using validated clinical cut-offs) is of utmost importance to detect potential self-related deficits. Here, we explore the potential use of Self-Other Voice Discrimination (SOVD) task for this purpose, as it is an experimentally validated marker for self-related deficits [[10], [11], [12]].

Given the intimate role self-voice plays in our identity, and that self-voice misperception has been related to psychotic symptoms such as auditory hallucinations [13], a specialized clinical tool integrating self-voice perception could be promising for detecting self-related deficits. To date, some studies have successfully attempted the objectivation (e.g., quantification) of voice recognition abilities within clinical neuropsychological examinations, by indicating specific brain regions and mechanisms which, if altered, can lead to a voice recognition disorder known as phonagnosia, which is described in neuropsychology as a voice recognition disorder, in the absence of name or face recognition deficits [14]. Several studies on healthy subjects have demonstrated selective voice perception mechanisms, highlighting distinct brain regions involved in distinguishing familiar from unfamiliar voices, particularly in the right temporal and frontal lobes, as shown in seminal PET research [15]. Other studies have expanded this investigation by including the perception of one's own voice compared to familiar or unfamiliar voices, with fMRI experiments consistently showing the role of the right hemisphere [16,17]. However, these studies have been limited in addressing self-related mechanisms due to their use of sentences or words/pseudowords as stimuli, which may engage broader neuropsychological functions [14]. Recent advancements have addressed this limitation by employing vocalizations in oddball paradigms methods [18,19], by adopting equalization procedure of auditory stimuli to minimize pre-attentive processing of self-generated and non-self-voices [20], or adopting continuous stimulus presentation to target the early voice perception effects [20]. The integration of EEG monitoring in such studies has enabled the detection and confirmation of frontal and temporal localization (electrodes) critical for discriminating self-voice specific stimuli around 300 ms post-stimulus. However, single-channel EEG analysis overlooked the insights from using all brain electrodes to better target the brain regions implicated. Moreover, these studies often failed to consider the physical aspects of voice perception which happens also through bone conduction [21].

Recently, we introduced the Self-Other Voice Discrimination (SOVD) task, utilizing neutral and equalized vocalizations, high-density EEG, and bone-conducting earphones. This approach aimed at combining subjective perceptual experiences with objective neuronal mechanisms involved in distinguishing one's own from others' voices [9]. In validation phases with healthy subjects, the SOVD task identified a specific brain network — the self-voice network — involving the right insula, cingulate cortex, and medial temporal lobe structures. This network's activation correlated with the accuracy of self-voice identification [10]. Importantly, the SOVD task has demonstrated clinical relevance by identifying personality alterations following neurosurgical interventions. For instance, in a patient undergoing resection and ligation of the superior sagittal sinus due to a meningioma (i.e., disrupting the self-voice network) led to impaired performance in the SOVD task post-surgery, with confusion between self and other voice conditions correlating with psychiatric symptoms and borderline personality disorder incidence [11]. Similarly, clinical studies utilizing self-voice tasks have investigated patients with stroke and healthy controls, demonstrating that right hemisphere lesions impair voice discrimination, whereas lesions in either hemisphere impair voice identity recognition [22,23]. However, previous clinical studies do not provide any clinical value/cut-off for categorizing a patient's performance as normal or deficient, in comparison with expected performance in HC. In other words, these studies lack a dedicated analysis allowing to categorize the individual performance and assess the prevalence of self-related deficits.

In line with the methodology followed to characterize the phonagnosia, the aim of the current exploratory study was to evaluate the discriminatory potential of the SOVD task to detect specifically self-voice related deficits, in terms of standardized psychometric measures with analyses at individual level. To that purpose, 17 neurosurgical patients, prior to their neurosurgical intervention, were enrolled in a SOVD task and their performance was analyzed in comparison to standardized data obtained from a comparable group of HC participants.

Based on these considerations, we hypothesized that the SOVD task would show a clinical discriminatory potential based on patients’ performance in Self-voice trials as compared to the Other-voice trials.

## 2 Methodology

### 2.1 Participants

**Fig. 1:**
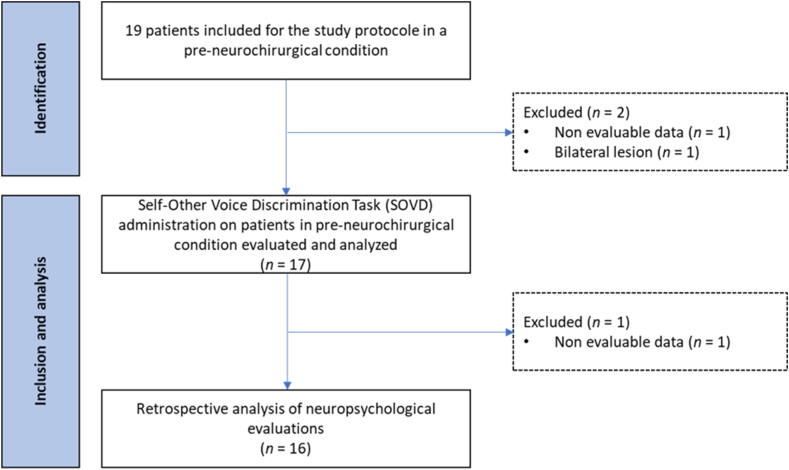
Flowchart of the study.

We included 17 patients (5 females; M_age_ = 46.17, SD_age_ = 14.94, range = 18–69 years; socio-cultural level II [compulsory post-school education, including apprenticeship and diploma] to III [compulsory post-school and higher education]); all patients were right-handed; 10 right-hemisphere lesions and 7 left-hemispheric lesions) at the Geneva University Hospitals (HUG) suffering from a focal hemispheric brain tumor (15 patients) or having a focal epileptic lesion (2 patients), who were candidates for neurosurgery (see Table 1). Based on a power analysis involving the comparison of two means ($N=\frac{2\times \sigma^{2}\;\left(z_{\frac{\alpha }{2}}+z_{\beta }\right)\;}{{\left({\overset{®}{X}}_{1}-{\;\bar{X}}_{2}\right)}^{2}}$), processed by BiostaTGV (https://biostatgv.sentiweb.fr/) [24] and carried out on a study that evaluated self-voice in patients with stroke [22], a sample of 15 patients was considered sufficient to achieve the (1 - β) of 90 % and a risk of Type I error (α) of .05, in a two-sided hypothesis (see Fig. 1).

**Table 1:** Sociodemographic, clinical and neuropsychological characteristics of patients.

Patient	Clinical characteristics			Memory				Executive				Instrumental					Attention
	Affected hemisphere	Type of lesion	Localization	verbal episodic	V-S episodic	verbal short-term	V-S short-term	Program-ming	Mental flexibility	Inhibition	Verbal Working memory	V-S memory	Verbal fluency	Perception	Language prod.	Languag comp.	Attention
1	R	Oligoden-droglioma OMS 2	Insula and fronto-opercular pars opercularie	DEF	NOR	NOR	NOR	DEF	DEF	DEF	DEF	DEF	DEF	NOR	NOR	NOR	DEF
2	L	Glioma	Superior frontal gyrus and cingulate gyrus	NOR	NOR	NOR	NOR	NOR	NOR	NOR	NOR	NOR	NOR	NOR	NOR	NOR	NOR
3	R	Astrocytome grade II	Temporo-mesial and insular	DEF	NOR	NOR	NOR	DEF	NOR	NOR	NOR	NOR	NOR	NOR	NOR	NOR	DEF
4	R	Astrocytome grade II	Middle frontal gyrus	NOR	NOR	NOR	NOR	NOR	NOR	NOR	NOR	NOR	NOR	NOR	NOR	NOR	NOR
5	L	Glioblastome	Temporo-occipital junction	DEF	NOR	NOR	NOR	NOR	DEF	NOR	NOR	NOR	NOR	NOR	DEF	NOR	DEF
6	R	Glioblastoma	Insula	DEF	NOR	NOR	NOR	NOR	NOR	NOR	NOR	NOR	NOR	NOR	NOR	NOR	NOR
7	R	Glioblastoma	Hippocampus and temporo-parietal	NOR	NOR	NOR	NOR	NOR	NOR	NOR	DEF	NOR	DEF	DEF	NOR	NOR	DEF
8	L	Astrocytoma grade II	Superior frontal and anterior gyrus	NOR	NOR	NOR	NOR	NOR	NOR	DEF	NOR	NOR	NOR	NOR	NOR	NOR	NOR
9	R	Glioblastoma	fronto-opercular pars opercularis and triangularis	DEF	DEF	NOR	NOR	NOR	DEF	NOR	NOR	NOR	DEF	NOR	NOR	NOR	DEF
10	R	Meningioma	Frontal	NOR	NOR	NOR	NOR	DEF	NOR	NOR	DEF	NOR	NOR	NOR	DEF	NOR	NOR
11	L	Ganglioglioma	Superior temporal gyrus	NOR	NOR	NOR	NOR	NOR	NOR	NOR	NOR	NOR	DEF	NOR	DEF	NOR	NOR
12	R	Astrocytome grade II	Hippocampus	NOR	NOR	NOR	NOR	NOR	NOR	DEF	NOR	NOR	NOR	NOR	NOR	NOR	DEF
13	L	Epilepsy	Fronto-temporal	NOR	NOR	NOR	NOR	NOR	NOR	NOR	NOR	NOR	NOR	NOR	NOR	NOR	NOR
14	R	Astrocytoma grade II	Frontal	DEF	NOR	NOR	NOR	DEF	NOR	DEF	DEF	NOR	DEF	NOR	DEF	NOR	NOR
15	L	Epilepsy	Fronto-temporal	DEF	DEF	NOR	NOR	DEF	DEF	NOR	NOR	NOR	NOR	NOR	DEF	NOR	DEF
16	L	Metastasis	Fronto-opercular	DEF	NOR	NOR	ND	DEF	NOR	NOR	NOR	ND	DEF	DEF	NOR	NOR	DEF
17	L	Glioblastoma	Parietal lobule	ND	ND	ND	ND	ND	ND	ND	ND	ND	ND	ND	ND	ND	ND

**Legend.** DEF: Deficit for the neuropsychological function evaluated (<−1.00 Z-scores; < Percentile 16; <40 T-score); F: Female; L: Left; M: Male; NOR: Normal (>−1.00 Z-scores; > Percentile 15.99; >39.99 T-score) neuropsychological performance; R: Right; V-S: Visuo-spatial.

To define clinical psychometric values, we extracted data from 17 HC (9 females; M_age_ = 37.29; SD_age_ = 15.58) from a previous study [10].

All participants reported no hearing deficits. Moreover, participants’ clinical history was evaluated and no psychiatric condition anterior to the evaluation was objected. All participants gave their informed consent prior to their participation and received monetary compensation (CHF 20/h).

The study was conducted in accordance with the Declaration of Helsinki and received approval from the Commission Cantonale d'Ethique de la Recherche sur l'être humain (CCER, code approved protocol PB_2016-01635).

## 3 Data availability

All data produced in the present study are available upon reasonable request to the authors.

### 3.1 Neuropsychological assessment

Retrospectively, we extracted the neuropsychological examinations (see Table 1) performed by board-certified neuropsychologists for all patients except one (the neuropsychological assessment could not be retrieved) in the context of the pre-surgical clinical assessment in an outpatient, non-hospitalized setting for 14 patients and 2 patients during their hospitalization. Nevertheless, since neuropsychological assessment was not part of the initial experimental design, the neuropsychological evaluations were not standardized for the type of tests used to assess different neuropsychological functions. As an example, different tests were performed for verbal episodic memory [e.g., MEM-III [25], RL/RI16 [26] or 15 words from Rey]. However, for all evaluated neuropsychological functions (memory; executive; attentional; instrumental [language; gestural praxis; visual gnosis]), psychometric data allowed to determine the presence or absence of cognitive deficits (see Table 1). For language, the majority of assessments were carried out for production abilities (e.g., via oral object naming tasks), while comprehension was tested on the basis of clinical assessment of boards-certified neuropsychologist. In case of doubts about language abilities, a team of speech therapists had the possibility of carrying out an additional speech therapy assessment, which was not the case for the 16 patients evaluated in the context of this study. Based on all these considerations and in order to compensate for the methodological biases, we dichotomized the performances for the neuropsychological functions as NORMAL/DEFICITARY, based on the normative data of the different neuropsychological functions carried out. This allowed to make to include the different neuropsychological functions in the analysis models. It is important to note that, the dichotomization (NORMAL/DEFICITARY) of neuropsychological performances was based on the standards established by the Swiss Association of Neuropsychology (ASNP) allowing an interpretation of standardized data [27,28]. A performance was considered “below standard” with the following standardized scores: z-score between −1.61 and −1.99; T-score between 30.01 and 33.50; Percentile between 2.01 and 5.00. A performance was considered “much substandard” with the following standardized scores: z-score < -2.00; T-score <30.00; Percentile <2. In order to avoid as many type I errors as possible, we chose a non-conservative threshold to categorize as deficient, therefore starting from a score considered to be “within the lower limit of the norm” (z-score < −1.00; T-score <40.00; Percentile <16.00). This dichotomization has been used previously [20] and the proposed methodology to categorize the prevalence of neuropsychological deficits has been validated on a Swiss population previously by our group [29,30].

### 3.2 Self-other voice discrimination task (SOVD)

Each patient participated in the SOVD task, following the procedure described in our previous works on HC [10]. Prior to the day of the experiment, patient's voice was recorded while vocalizing the/a/phoneme for approximately 1–2 s (Zoom H6 Handy recorder). Recordings were standardized and cleaned of background noise with Audacity software (−12 dBFS, 500 ms). Short vocalizations were chosen to control for variables like accent and prosody, and to focus on the voice's acoustic properties for speaker identification, as shown effective in Mary Zarate, Tian [31]. Each preprocessed vocalization was then mixed with a target voice of a gender-matched unfamiliar person to create voice morphs spanning a voice identity continuum, by using TANDEM-STRAIGHT (MATLAB package), based on interference-free speech representations, which decomposes recordings from two speakers into independent acoustic parameters that can be precisely controlled and interpolated [32]. In details, six voice-morphs were generated containing different percentage of patient's self-voice, namely: 15 %, 30 %, 45 %, 55 %, 70 %, 85 %.

During the experiment, patients were sat alone in a comfortable armchair in a dedicated silent and moderately lit room. The voice-morphs were presented in random order to the patient through laptop loudspeakers. Patients were asked to discriminate whether a heard voice-morph resembled Self and Other's voice. A total of 50 trials of each voice-morphs were presented with an inter-trial variability between 1 and 1.5 s.

### 3.3 Statistical analysis

First, patients' performances on the SOVD task was measured in terms of score of accuracy to detect self/other voice-morphs. Global score; Self-voice score; Other-voice score were extracted and Z-scored, based on HC performance as follows:

$$Z\_score\_patient=\left(\frac{\text{Patient}\;\_\;\text{score}-{Mean}_{HC\_score}\;}{{SD}_{HC\_score}}\right)$$

This standardization allowed to control for age and gender effects. The patient's z-scores were then categorized (deficit/non-deficit) according to the conservative clinical cut-offs established by the Swiss Association of Neuropsychology (deficit: < - 1.60 Z-score).

In order to exclude the potential effects of confounding factors, 6 distinct models of backward stepwise multiple regression were carried out: *i) for neuropsychological predictors*: between the standardized SOVD z-scores (i.e.: Self-voice; Other-voice; Global) and neuropsychological performances; ii) *for other clinical predictors*: between the standardized SOVD z-scores (i.e., Self-voice; Other-voice; Global) and secondary clinical variables (lateralization of lesions; type of lesion).

### 3.4 Data availability statement

All data produced in the present study are available upon reasonable request to the authors.

## 4 Results

### 4.1 Prevalence of deficits

No significant differences between patients and HC were found in terms of sociodemographic data.

**Fig. 2:**
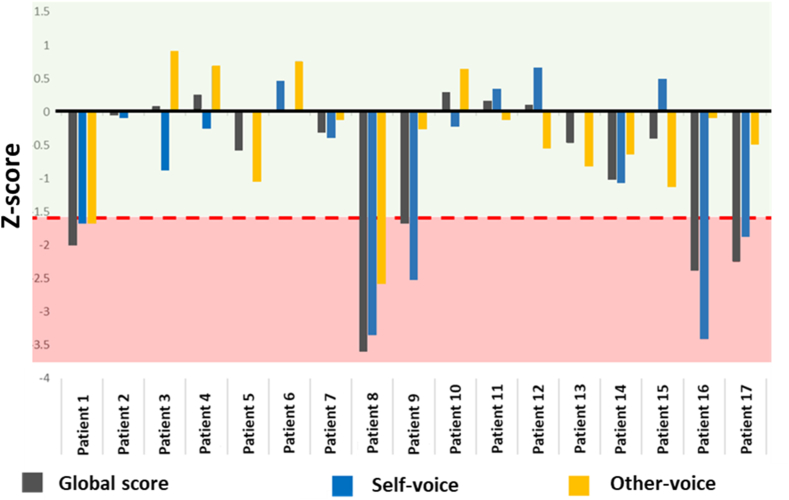
Z-standardized scores for the self-other voice discrimination (SOVD) task scores. Based on Swiss Association of Neuropsychology (ASNP) guidelines, a threshold of −1.60 Z-score (delimited by the red line) was set for the definition of impaired performance. impaired performances were observed for 5 patients for the global score (gray bars); 3/5 had a specific deficit of Self-voice recognition (Patients 9,16,17).

Analysis of standardized scores revealed that 5 patients (29.41 % of the sample) presented deficits in performance for the Global score on the SOVD task and 12 patients (70.59 % of the sample) displayed comparable performances (for all SOVD measures) to the HC group. By considering the specific conditions (Self-voice and Other-voice), only 2 out of the 5 patients (11.76 % of the global sample) presented an impaired score for the recognition of both the Self- and Other-voice, suggesting a general deficit in voice discrimination (e.g., phonagnosia). Interestingly, 3 patients (17.65 % of the global sample) had difficulties specific to Self-voice recognition, in the absence of other-voice or general voice recognition deficits, suggesting a potential self-related deficit (see Fig. 2).

### 4.2 Confounding factors

For the evaluation of *neuropsychological predictors,* we observed significant results only for the Other-voice condition performance, which was associated with the presence/absence of inhibitory deficits (*t* = 2.87, *p* = .013). All other predictors were non-significant (*all p's* > .051).

For the evaluation of *other clinical predictors* (i.e. hemisphere of lesion and type of lesion), analysis did not reveal significant results (*all p's* > .08).

## 5 Discussion

Here we conducted an exploratory analysis on a cohort of neurosurgical patients who were enrolled in a SOVD task, in the context of a previous research project. The aim was to evaluate, through standardized statistical analyses commonly used in neuropsychological psychometrics, the clinical discriminatory potential of the SOVD task for the objectification of self-related voice deficit. Although the majority of the patients considered in this work were comparable to HC in terms of task performance, the SOVD task allowed to discriminate distinct clinical phenomena. Indeed, among patients presenting impaired performance (5), it was possible to distinguish a subgroup of patients (2) likely affected by phonagnosia (impaired performance both for Self- and Other-voice discrimination) and a subgroup of patients (3) characterized by self-related deficits, manifested by impaired performance specific to the Self-voice recognition condition. These results reveal the potential of the SOVD task in targeting Self-voice impairments, independently from the presence of deficits in other neuropsychological functions assessed. In the case of recognition of the Other-voice condition, the presence/absence of inhibitory deficits was significantly associated with Other-voice trials, suggesting the involvement of executive processes in voice processing, as has already been demonstrated previously [33]. Other clinical variables, such as hemispheric lateralization of the lesion, type of pathology, and the presence of neuropsychological deficits were not predictive of the SOVD performance, in particular for the Self-voice condition. Brain hemispheric lateralization of self-voice is quite debated in the literature. This mainly depends on the use of different methodological approaches, for example the voice type (self, familiar, unfamiliar) and/or the characteristics of stimuli (sentences, words/pseudowords, phonemes). In an fMRI study with healthy subjects distinguishing between self and other voices pronouncing words, greater activation was observed in the left frontal gyrus and anterior cingulate gyrus in response to self-voice stimuli [17]. However, Kaplan, Aziz-Zadeh [16] found distinct activation patterns, specifically in the right inferior frontal gyrus, when subjects processed sentences pronounced by their own voice compared to a familiar voice. The most significant contributions derive from patient data. In a recent study involving a large cohort of patients with unilateral damage, Roswandowitz, Kappes [34] elucidated that specific structures within a broader right hemispheric network are indispensable. Indeed, they demonstrated that the right posterior/mid temporal lobe plays a crucial and obligatory role in recognizing one's own name when pronounced by one's own voice. Candini, Avanzi [22], who studied stroke patients, examined the performance of patients after stroke by presenting pairs of words pronounced in three different voice types (self, familiar, unfamiliar). Patients were tasked with: i) determining if their own voice was present among the stimuli (self-recognition task); and ii) discerning whether two voice samples were the same or different (self-voice discrimination task). In the self-recognition task, which closely parallels our study, both left and right hemispheric patients exhibited impairments compared to healthy subjects. Our results do not show a significant effect of brain lateralization. This seems to be consistent with the precedent work. However, in our opinion that the lack of significant findings may be influenced by statistical limitations in our regression analysis, despite balancing the sample with 9 right-hemisphere lesions and 9 left-hemisphere lesions. Similarly, this could have influenced the identification of effects related to specific localization. In the future, a large cohort of patients with more homogeneous sites and types of lesions will help in further detailing our findings. However, it is important to note that our current aim was to characterize the role of an experimentally validated task, SOVD, within an integrated neuropsychological assessment framework for distinguishing either voice and, in that context, self-voice impairments. Compared with previous studies [22], we adopted for patients the use of phonemes in order to minimize the potential involvement of high other cognitive functions (for example, at the level of semantic or executive memory) associated with the use of names word/pseudowords, and sentences.

Our current results underline the importance to integrate adapted experimental tools for assessing the sense of self in the clinical practice, emphasizing the relevance of self-voice-based tasks. In addition, an objective assessment of self-related impairments would enhance the comprehension of patients’ symptomatology and could be useful for personalized neuropsychological rehabilitation. Importantly, considering its short duration (∼15 min), the SOVD task could find practical applicability in the neuropsychological routine, across various pathologies. Indeed, the development of an objective tool for evaluating self-deficits not only allows a better evaluation of deficits in various pathologies, making it possible to go beyond the clinical evaluation by specialists who may be subject to bias [5]. But also, it can have important implications from an experimental point of view to define the brain regions and networks of interest, possibly making it possible to guide interventions from a cognitive point of view, for example in the neurosurgical context. Finally, this could also provide valuable information in the context of neurorehabilitation, in order not only to enable the development of remediation techniques (which could be monitored longitudinally with the use of SOVD), but also for rehabilitation. other cognitive functions.

Nevertheless, the experimental paradigm has several limitations, which in the future will need to be specifically addressed for a specifically dedicated psychometric validation of the SOVD task. First of all, increase the number of all participants (healthy controls and patients), in order to develop standards adapted to different socio-demographic variables (e.g., age; gender; socio-cultural level). Secondly, the inclusion of standardized neuropsychological assessment for the evaluation of associated factors, as for example an exhaustive evaluation of executive and attentional functions, with the objective of assessing the relationship widely described in the literature (mainly in the context of neurodegenerative pathologies) of executive-attentional functions with meta-cognitive functions, based on cognitive processes such as “an executive buffer” or via shared neural networks and this in the context of cognitive models (e.g., the Cognitive Awareness Model (CAM) [2,35,36]). Therefore, the inclusion of standardized and exhaustive neuropsychological evaluation would be important to encompass the limitations inherent to the neuropsychological assessments available for our sample, but would also make it possible to acquire knowledge of cognitive processes linking the self to other cognitive functions, potentially opening the way to the implementation of compensatory rehabilitation processes [2]. Thirdly, the neuropsychological assessments were retrospectively extracted from the clinical environment and therefore lacked of a standardization in specific experimental terms. Although these assessments were carried out by board-certified neuropsychologists using validated and psychometrically standardized neuropsychological tools, there was heterogeneity in the tools used, thus limiting the possibilities of analyzing the data in a continuous manner. This was particularly true for the assessment of language abilities. Although production abilities were specifically evaluated through validated psychometric tools, comprehension abilities were based on clinical assessments. However, we applied statistically valid methods to partially compensate for these biases, which in the future will need to be considered in the development of validation protocols. Fourth, although possible auditory impairments do not affect the SOVD performance (given the counterbalancing of conditions), it would be useful to integrate explicit analysis for auditory performance (e.g., pitch recognition). Finally, in addition to the present limitations, to better characterize the mechanisms inherent to voice-identity recognition, it would be important to develop the SOVD task by contrasting vocal stimuli pronounced by self/familiar and unfamiliar voices [14].

In conclusion, this work shows that the SOVD task could serve as a psychometrically validated tool in the clinical setting for an objective and standardized neuropsychological assessment of self-related deficits.

## Funding

This research was supported by the 10.13039/501100001711Swiss National Science Foundation (grant no. 320030_182497 to K.S.).

## CRediT authorship contribution statement

**Philippe Voruz:** Writing – original draft, Visualization, Resources, Methodology, Investigation, Formal analysis, Conceptualization. **Pavo Orepic:** Writing – review & editing, Resources, Methodology, Investigation, Conceptualization. **Selim Yahia Coll:** Writing – review & editing, Resources, Investigation, Formal analysis, Data curation. **Julien Haemmerli:** Writing – review & editing, Resources, Data curation. **Olaf Blanke:** Writing – review & editing, Resources, Methodology, Investigation. **Julie Anne Péron:** Writing – review & editing, Validation, Supervision, Methodology. **Karl Schaller:** Writing – review & editing, Validation, Supervision, Resources, Methodology, Investigation, Funding acquisition, Conceptualization. **Giannina Rita Iannotti:** Writing – review & editing, Validation, Supervision, Resources, Project administration, Methodology, Investigation, Funding acquisition, Data curation, Conceptualization.

## Declaration of competing interest

None of the authors has declared an interest.
